# Post-Traumatic Stress Disorder in the Military Police of Rio de Janeiro: Can a Risk Profile Be Identified?

**DOI:** 10.3390/ijerph18052594

**Published:** 2021-03-05

**Authors:** Fernanda Dias Campos, Maria José Chambel, Sílvia Lopes, Paulo C. Dias

**Affiliations:** 1 Military Police of the State of Rio de Janeiro, Rio de Janeiro 20031-040, Brazil; fernandacampos@edu.ulisboa.pt; 2 CICPSI, Faculdade de Psicologia, Universidade de Lisboa, 1649-013 Lisboa, Portugal; mjchambel@psicologia.ulisboa.pt (M.J.C.); silopes@ucp.pt (S.L.); 3 Centre for Philosophical and Humanistic Studies, Faculty of Philosophy and Social Sciences, Universidade Católica Portuguesa, 4710-302 Braga, Portugal

**Keywords:** post-traumatic stress disorder, military police, socio-demographic, occupational variables

## Abstract

Background: Significant exposure to critical incidents characteristic of military police work has a potentially traumatic effect and multiple consequences for the mental health of these professionals, such as Post Traumatic Stress Disorder (PTSD). This study aims to investigate the occurrence of PTSD in this occupational group and its correlations with socio-demographic and occupational variables. Methods: This is a cross-sectional study of Rio de Janeiro’s Military Police officers (*n* = 3.577). Data was collected from self-reported questionnaires applied in an institutional health program. Post-Traumatic Stress Disorder Checklist—Civilian version was used to assess PTSD. Results: Rates of 16.9% for full PTSD and 26.7% for partial PTSD were found. Based on logistic regression analysis, female officers and police officers in lower ranks of the military hierarchy and performing administrative duties were found to be at most risk of developing PTSD. Conclusions: These results suggest the need to further understand the predictive organizational and individual variables of PTSD correlated with the increased vulnerability of professionals in order to contribute to institutional policies for the prevention and rehabilitation of these cases.

## 1. Introduction

Post-traumatic stress disorder (PTSD) is a condition resulting from direct or indirect exposure to traumatic events that pose a risk to one’s life and physical integrity [1]. In fact, many references to the disorder found in the literature are based on clinical observations of symptoms presented by military personnel in the wake of major conflicts [2,3]. The inclusion of the disorder in psychiatric nosology represents an acknowledgment of mental illness as a result of a high-impact critical threatening experience [4], and its symptoms include re-experiencing the traumatic event, persistent avoidance, negative changes in cognitions and mood and autonomic hyperstimulation, often accompanied by sleep problems or self-destructive behavior [1].

Police officers constitute an occupational group that is repeatedly exposed to critical incidents, thus placing them at greater risk of developing PTSD [5,6,7,8]. In their daily work, they encounter both violent incidents in which they experience trauma directly (i.e., shootings, ambushes, riots) and depressing incidents, which require direct professional action in the aftermath of violent events, such as assisting victims of accidents and physical and sexual assaults and contact with corpses [5]. Weiss et al. [9] showed that out of 719 American police officers, 50.8% had been threatened with weapons, 55.2% with knives, 82.2% had been involved in high-speed vehicle chases, 20.3% had had a colleague killed on duty, 40.2% had attended an incident with a child who had been sexually abused, and 87.2% had witnessed the death of a person directly. In addition, 15.9% of all the police officers reported having been seriously attacked while on duty at least once in their professional careers.

Studies on PTSD among police officers have pointed to a significant impact on the mental and physical health of the affected professionals, in addition to the impairment of their social and professional functioning [10]. Police officers who have been exposed to traumatic events present more comorbid psychological conditions, including depression, suicidal ideation, substance abuse, and anxiety [7,11], and lower levels of job satisfaction [12]. All these symptoms present a potential risk for the police activity and directly affect security policies.

Among police officers, a PTSD prevalence of 7% to 19% has been found [5,6,8,13,14,15], with higher rates for those with subsyndromal/partial PTSD. It is important to note that researchers observed that some cases, even without presenting the complete set of symptoms for PTSD, also seemed to suffer from clinically configured symptoms of the disorder. Thus, the concept of partial PTSD was defined for its subsyndromic forms, by presence of some, but not all symptoms required. For example, some authors, [16,17] established that at least one symptom in each DSM-IV category was necessary to qualify as partial PTSD. Among Brazilian police officers, Maia et al. [6] found a PTSD prevalence of 8.9% and a partial PTSD rate of 16% in military police officers of an elite military police unit of the Goiás State.

Variations in prevalence rates may be explained by socio-demographic factors, specific regional features (local levels of violence), different evaluation methods, the socio-economic conditions of the professionals and the population they serve, and the working conditions that support police action. Additionally, there are individual and organizational variables that may contribute to higher or lower PTSD rates, which indicates that the understanding of the phenomenon in different police corps is dependent on broader knowledge of the various risk and prevention factors associated with the specific nature of each organization.

### 1.1. Demographic and Occupational Characteristics and PTSD in Police Officers

According to Marchand et al. [18], research has focused on several socio-demographic factors and their connection to PTSD in the general population (age, gender, race/ethnic group, and schooling). However, overall literature has not been conclusive on how these variables impact vulnerability to post-traumatic symptoms. Moreover, police officers clearly differ from other occupational groups in many respects, and in order to understand the PTSD phenomenon in this group, it is essential to analyze the specific factors related to this professional activity.

Contrary to what is commonly found in the general population, many studies have shown that female police officers do not appear to be at any greater risk of developing PTSD than their male counterparts [18,19,20,21]. In line with these findings, in a systematic review of the literature on the prevalence of PTSD in rescue workers (police officers, fire-fighters, and emergency professionals), Berger et al. [22] found no association between gender and PTSD for these populations. The authors stress that it may be assumed that some of the characteristics of military groups, such as rigorous selection criteria and training, may narrow gender differences in terms of the risk of PTSD. However, they highlight that in 75% of the reviewed studies, approximately 85% of the samples consisted of males, which may reduce the possibility of adequately detecting the effect of this variable on PTSD in these groups.

On the other hand, the studies that found significant correlations between the gender variable and PTSD in police officers indicated that its predictive role for the disorder may vary according to the type of traumatic incident experienced and its frequency. Females are usually more susceptible to sexual abuse than males, who are the target of more personal attacks [18]. This might suggest that the type of trauma experienced may be more predictive of the illness than gender itself. In the same vein, Surgenor et al. [23] highlight that as the female status is associated with higher levels of PTSD in some studies, this suggests that cultural and economic differences may influence this connection, since these aspects may reflect greater or lesser exposure to gender-based violence and other risk factors to mental health, such as lack of social support, sexual, and moral harassment or social vulnerability.

The research on schooling and marital status are even scarcer. As far as schooling is concerned, studies with rescue workers highlight that lower levels of schooling [20,24] and lower levels of training [25,26] may be risk factors for PTSD. Regarding marital status, in a study with Brazilian police officers from the state of Goiás, divorced respondents were found to present a five-fold higher risk of PTSD [6].

According to several studies [19,27,28], the age variable has not been found to be correlated with higher levels of PTSD symptoms. On the contrary, other studies have indicated that this variable affects vulnerability to the disorder, as in the case of younger police officers in the early stages of their career. This factor has a higher correlation with PTSD, especially for those who are confronted with critical incidents for the first time [29,30]. It should be noted that this variable may be influenced by a lack of experience in the operational context. In fact, studies have shown that reduced work experience has increased the risk of PTSD among young police officers [30,31].

In addition to the demographic factors, research should also focus on the occupational features and their impact on the post-traumatic outcome, since different ranks and positions of the institutional hierarchy, seniority within the institution (which is reflected in the years of experience), and the different duties performed by the police officers (i.e., ostensive foot or motor patrol, tactical police operations, civil disorder control) offer a context of higher or lower exposure to critical incidents, according to their specific features.

Few studies have addressed the correlations between PTSD and other occupational variables such as position in the institutional hierarchy, and the duties performed by police officers. In a study with Korean police officers, Lee et al. [32] observed that the Assistant Inspector Group was the hierarchical rank that showed higher rates of PTSD (42.7%), and the Intelligence and National Security Division was the work department that showed the highest frequency for the disorder, suggesting that working in different divisions was associated with the prevalence of PTSD.

Likewise, in a study with the Canadian public safety personnel (PSP), Carleton et al., [7] found significant differences across categories with respect to mental disorder rates. The Royal Canadian Mounted Police (RCPM), correctional workers, and paramedics were generally more likely to experience all mental disorders, including PTSD, when compared with municipal/provincial police. Similarly, Di Nota et al. [33] observed that RCMP officers reported more suicidal ideation than other police officers and scored highest on measures of PTSD, depression, anxiety, stress, and panic disorder. This may indicate that the characteristics of the duties performed in each category can predict occupational risks to mental health.

On the other hand, in a study in a Swiss state police department with officers from the criminal, community, and emergency divisions, Habersaat et al. [34] observed that although community officers reported more posttraumatic stress symptoms, the results appeared to be more dependent on personal factors and individuals’ perception of work conditions than division-specific work environments.

Moreover, although these studies have addressed the correlations between PTSD and the role played by practitioners and the department in which they operate, their findings, as a result of the observable cultural differences among the samples under study, may not be easily extended to other contexts, especially to the Military Police of Rio de Janeiro, since its role and organizational structure are considerably different.

### 1.2. Military Police Officers of Rio de Janeiro—Brazil

In Brazil, “the military police officers are responsible for ostensive policing and the preservation of public order” [35]. Thus, these officers perform uniformed policing 24 h per day in urban roads, focusing on crime prevention as their priority. Their action includes a wide variety of situations such as approaching, immobilizing, and arresting criminals or offenders, surrounding and pursuing suspect vehicles, seizing weapons, drugs, and illicit material and assisting members of the public.

The state of Rio de Janeiro presents specific features compared to other regions of the territory as far as the criminal dynamics observed in Brazil are concerned. The public security policy of the state is characterized by the repression of heavily armed groups associated with the illegal trafficking of weapons and drugs, which operate from geographically challenging locations that hinder easy access by the police teams. These teams are also sometimes called upon to intervene in armed confrontations between rival groups. Consequently, these professionals are often exposed to highly volatile environments, where ambushes, shootings, the rescue of fellow officers, and physical attacks (both as perpetrators and victims) are occurrences that frequently result in the injuries and deaths of police officers and citizens.

Although the military police officers in the state of Rio de Janeiro are often exposed to this variety of critical events in their daily work, the incidence and predictors of PTSD are still unexplored in this population. This study seeks to contribute to filling this gap in the literature by investigating the risk profile for PTSD among these professionals. It is expected to contribute to refining appropriate institutional policies for the prevention of mental illness at an organizational level, and also to the field of occupational mental health research applied to public security in Brazil.

### 1.3. The Present Study

On December 2016, the Directorate of Health of the Military Police (DGS), through the Central Unit of Psychology (NUCEPSI), implemented PTSD screening for active military police officers within the scope of the general health assessment of the Police Health Care Service (SASP). The SASP consists of an institutional health program that focuses on disease prevention through the early identification of these professionals’ health problems. To accomplish this goal, police officers in active service in the Corps undergo multidisciplinary health assessments.

Based on the assessments made within the scope of the SASP program, this study seeks to investigate the incidence of PTSD and partial PTSD among these professionals by establishing its association with demographic (gender, age, marital status, and schooling) and occupational (working hours, tenure, position in the military hierarchy, and duties) variables. Its relevance is grounded in the assumption that the performance of prevalence studies and diagnoses in professional categories contributes to shedding light upon the working conditions that may lead to certain occupational comorbidities.

Considering the studies conducted in Brazil and the rates of violence observed in Rio de Janeiro compared to other states, higher rates of PTSD and partial PTSD than in the remaining states of the country and international studies are expected to be found. As far as the demographic features are concerned, this study assumes that gender (female), marital status (divorced), and age (younger police officers) may be related to PTSD among this population. Additionally, it is believed that some duties (Tactical Policing—GAT—employed when a qualified response in areas of criminal imbalance is required, which include specific operations, like detention of criminals and seizure of illicit materials, with greater use of lethal force and higher risks of resulting in the death or injury of an agent), types of units (Pacifying Police Units (UPPs), community police units, usually located in underprivileged communities, some at risk of armed conflict between marginal groups), position in the hierarchy (soldiers), and a lower level of seniority will have a higher significance in predicting PTSD, as these features offer greater exposure to the operational risk.

## 2. Materials and Methods

This cross-sectional study used data from the records of police officers’ health assessments carried out by the Police Health Care Service (SASP), between 2017 and 2019. These professionals performed operational functions (ostensive and regular foot or motor patrol, specialized tactical policing, police intelligence activities) or administrative functions (secretariat, treasury, logistics, personnel control, etc.).

The officers were first given a brief lecture in which SASP health professionals explained the program’s objectives and procedures. The police officers were then instructed on how to fill in the health forms (nutritional information, health habits, pre-existing health conditions, etc.) and inventories that were part of the psychological assessment. They were then individually interviewed by the psychologists who analyzed the inventories and their scores and referred the police officers to individual specialized care at one of the corps’ health units when significant mental suffering was detected. Finally, the interviewed officers signed an informed consent form stating they had understood the guidelines received by the health professionals and that the results obtained would be anonymized in order to build a database on Rio de Janeiro’s police force.

Upon completion of the assessment, the police records were stored. Occupational and demographic data were registered on an anonymous database, along with the scores obtained from the inventories for analysis related to the objectives of the SASP program. The ethical aspects that ensure the confidentiality of the personal details of the assessed police officers were taken into consideration for the development of this study. Use of the data from the forms of the police officers assessed by the SASP for scientific publication was granted by the General Staff of the Military Police of Rio de Janeiro (EMG/SEMP—SEI-350503/000837/2020).

Socio-demographic and occupational data was collected with a questionnaire developed by SASP that included gender, age, marital status, qualifications, position/rank, duties performed (special tactical policing/reserved service/regular policing and radio patrol/administrative work/other), and tenure.

To test for PTSD, the Post-Traumatic Stress Disorder Checklist/Civil Version (PCL-C) was used. This inventory was developed by Weathers et al. [36], with semantic validity for Brazilian Portuguese at the beginning of the assessment [37], and extensively used in studies involving police officers [6,10,11,19,27,38]. It is a generalizable instrument with satisfactory psychometric characteristics in terms of validity and reliability [10,39].

The PCL-C scale is composed of 17 items and respondents are asked to measure the extent to which they have been affected by the symptoms described in the last month on a Likert type scale, where 1 corresponds to “not at all” and 5 to “very much,” according to DSM-IV criteria [40]. For a full PTSD result, the scores were sub scaled by symptoms [6,36]: an intensity response above or equal to three in at least one item of criterion “B” (re-experiencing), three items of criterion “C” (avoidance and numbing), and two items of the criterion “D” (hyperarousal). For the definition of partial PTSD, the existence of at least one symptom in each of the criteria was used [16,17]. Satisfactory internal consistency coefficients were found for total scale (α = 0.93) and for the three dimensions analyzed (α = 0.89 for Criterion B, α = 0.86 for Criterion C, and α = 0.77 for Criterion D).

The Statistical Package for the Social Sciences version 26—SPSS software –was used for the analyses. In the first stage, rates of PTSD, partial PTSD, and police officers without PTSD were estimated. Statistically significant differences between the proportions of categorial variables for the three groups created by the PCL scores were identified using Pearson’s χ2 statistic and we took *p* values of less than 0.05 to indicate statistical significance. Forward logistic regression was used to select the variables that significantly predicted whether a police officer was a full PTSD or partial PTSD case. B (unstandardized regression coefficient) and CI95 (confidence interval) were computed and correlations were considered statistically significant when *p* ≤ 0.05.

## 3. Results

The sample initially consisted of 3577 military police officers on active duty. After the withdrawal of the missing cases, a sample of 3.554 was left, more specifically 3316 males (93.3%) and 238 females (6.7%), with a mean age of 38 years (SD = 6.7) and an average of 12 years in the corps (SD = 7.1). Among the sample, a majority of the participants were aged between 30 and 39 years (51.5%) and 40 and 49 years (36.7%), followed by those aged between 21 and 29 years (8.6%), and a minority over the age of 50 years (3.3%). A large part of the sample was married (69.0%), 21.7% single, and a minority were divorcees (6.7%) and widowers (4%). More than half of the sample had completed 12 years of education (63.0%), followed by those with incomplete (14.8%) and complete (19.1%) higher education.

When observing the occupational characteristics of the sample, the considerable number of police officers occupying lower rank positions (53.9% soldiers and corporals and 37.2% sergeants) is noteworthy; 5.8% were sub-lieutenants, and 3.1% officers (lieutenants, captains, majors, and colonels), the higher ranks responsible for commanding positions in the military structure; 1.0% of the participants were observed to have more than 30 years of service in the military police, 13.6% had between 20 and 29 years, 34.4% between 11 and 19, 28.5% between 6 and 10 years, and 22.6% had less than 5 years in service. In the sample, 15.3% of police officers exercised tactical policing (GAT/PATAMO), 2.4% performed intelligence activities, 28.3% performed ordinary policing (PO/APREV/RP), 16.6% performed administrative duties, and 37.3% claimed to perform some other type of duty. The work schedules adopted in the organization varied, with daily schedules (Monday to Friday) normally adopted for administrative duties (21.5% of the participants), and different schedules adopted for operational duties, which alternated between 12 and 24-h work shifts with days off. In this sample, the most frequently adopted schedules were 12 × 24/12 × 48 (28.2%), 24 × 72 (21.4%), 24 × 48 (13.9%) and 12 × 36 (11.1%).

The analyses with this sample pointed to an incidence of 16.9% of the interviewed police officers with total PTSD and 83.1% without the disorder. For partial PTSD, 26.7% of the police officers reported the subsyndromal form of PTSD, indicating a group at risk of future illness. The prevalence rates for both probable and non-probable PTSD cases and the distribution of demographic and occupational variables (independent variables) according to each group are presented in Table 1 and Table 2.

Firstly, it should be noted that gender was a significant predictor to explain the risk of PTSD in this sample. As observed in the logistic regression, females appeared to be more vulnerable to the disorder than males, both to partial (B = −0.516, *p* < 0.01, IC 95% (0.424, 0.840)) and full PTSD (B = −0.485, *p* < 0.01, IC 95% (0.423, 0.896)). Likewise, a significantly higher proportion of females with full PTSD (χ2 (df = 1, N = 3554) = 27.72; *p* < 0.01) and partial PTSD (χ2 (df = 1, N = 3554) = 31.86; *p* < 0.01) was observed in comparison with males.

Although the proportions of age in the full PTSD group (χ2 (df = 3, N = 3554) = 8.61; *p* < 0.05) and in the partial PTSD (χ2 (df = 3, N = 3554) = 10.10; *p* < 0.05) were significantly different compared with the group without PTSD, age did not emerge as a predictor for full PTSD (B = −0.126, *p* > 0.01, IC 95% (0.400, 1.944)) and partial PTSD (B = −0.351, *p* > 0.01, IC 95% (0.742, 2.717)). Similarly, even though the police officers’ qualification appeared to have a different proportion in the full PTSD (χ2 (df = 6, N = 3554) = 22.60; *p* < 0.01) and in the partial PTSD (χ2 (df = 6, N = 3554) = 18.76; *p* < 0.01) groups compared with the group without PTSD. In the logistic regression analysis level of schooling, there was not a significant predictor of full PTSD (B = −0.518, *p* > 0.01, IC 95% (0.048–7.347)) or partial PTSD (B = −0.168, *p* > 0.01, IC 95% (0.065–11.014)).

Marital status did not appear to be related to the disorder in any of the analyses performed, and an identical distribution was observed in the group without PTSD and in both the full PTSD (χ2 (df = 4, N = 3554) = 5.05; n.s.) and partial PTSD (χ2 (df = 4, N = 3554) = 6.24; n.s.) groups. Moreover, concerning the logistic regression analysis, this variable was not a significant predictor of full PTSD (Wald = 2.793, *p* = 0.593) or partial PTSD (Wald = 2.410, *p* = 0.661).

Regarding the occupational variables, although the proportions of police tenure in the full PTSD group (χ2 (df = 6, N = 3554) = 15.06; *p* < 0.05) and in the partial PTSD group (χ2 (df = 6, N = 3554) = 13.10; *p* < 0.05) were significantly different compared with the group without PTSD, in the logistic regression analysis tenure was not a significant predictor of full PTSD (B = 0.665, *p* > 0.01, IC 95% (0.484–7.812)), or partial PTSD (B = 0.145, *p* > 0.01, IC 95% (0.361–3.697)).

Conversely, the hierarchical positions were observed to be an important predictor of PTSD, such as soldiers and corporals (B = 1.707, *p* < 0.01, IC 95% (1.997,15.229); B = 1.999, *p* < 0.01, IC 95% (3.086–17.648)), sergeants (B = 1.359, *p* < 0.01, IC 95% (1.436,10.557); B = 1.820, *p* < 0.01, IC 95% (2.610–14.599)) and sub-lieutenants (B = 1.377, *p* < 0.01, IC 95% (1.382, 11.299); B = 1.569, *p* < 0.01 IC 95% (1.930–11.951)) predicting full and partial PTSD, respectively. Moreover, the proportion of police officers with different hierarchical positions was significantly different for full (χ2 (df = 5, N = 3554) = 15.17; *p* < 0.01) and partial PTSD (χ2 (df = 3, N = 3554) = 21.57; *p* < 0.01), compared with the group without PTSD.

As far as the duties performed are concerned, the risk of PTSD appears to be higher in administrative professionals both for the development of full (B = 0.417, *p* < 0.01, IC 95% (1.085, 2.124)) and partial PTSD (B = 0.518, *p* < 0.01, IC 95% (1.245–2.265)). In contrast, professionals with GAT/PATAMO duties, corresponding to tactical policing activities in urban areas, appear to be significantly less likely to suffer from full (B = −0.507, *p* < 0.01, IC 95% (0.406–0.892)) and partial PTSD (B = −0.345, *p* < 0.01, IC 95% (0.521–0.963)). Moreover, in the group comparison analysis, the duties performed also appeared to significantly predict (χ2 (df = 5, N = 3554) = 123.71; *p* < 0.01; χ2 (df = 4, N = 3554) = 141.02; *p* < 0.01, full and partial PTSD, respectively).

Finally, the work schedule also appears to be a relevant factor to explain the incidence of PTSD. This variable was a significant predictor of full PTSD (Wald = 25.015, *p* < 0.01) and partial PTSD (Wald = 33.400, *p* < 0.01), no significant differences were found between the different schedule categories considered in full PTSD. On the other hand, the 24 × 72 schedule was observed to have a positive impact on partial PTSD (B = −0.995, IC 95% (0.139–0.983)).

## 4. Discussion

Based on the analyses, it was possible to ascertain that the PTSD rate among military police officers in Rio de Janeiro is in line with those already reported in the literature. However, when compared to international studies, it is higher than the rates found among Dutch police officers (7%) [5], American police officers, both after the World Trade Center terrorist attack (8.8%) [31], (12.9%) [19] and in policing duties (13%) [14]. However, the rate is lower than that found among US police officers after Hurricane Katrina in the USA (19%) [15], an event in which professionals not only rescued and offered support to the victims of the natural disaster but also suffered personal losses.

When compared to other Brazilian police institutions, the rates found in this study are significantly higher than, for example, those found in the military police officers of an elite corps in the state of Goiás, in which an 8.9% PTSD rate and 16% partial PTSD rate were observed [6]. Such disparity may be due to the sample, as specialized police groups tend to report less mental disturbance as a result of more training, greater team cohesion, better equipment, and greater professional recognition.

Regarding the subsyndromal form of the disorder, the finding of 26.7% indicates that just over a quarter of the sample presents not only a risk condition for future worsening of the syndrome but also a significant current impairment. In fact, although frequently ignored in clinical settings, partial PTSD may be chronic [41] and associated with high rates of other psychiatric disorders, as well as with functional difficulties [6,41,42]. The rate of active police officers who present the subsyndromal form of the disorder demonstrates the need for the implementation of specific psychological assessment programs aimed at identifying PTSD in the early stages after critical incidents. These programs should also take into account the typical re-exposure of police activity and foster early interventions to prevent a worsening of the syndrome.

As for the analyses of the predictive role of demographic and occupational variables for PTSD addressed in this research, it was possible to establish a risk profile for PTSD among the observed population. First, the highest level of vulnerability to the disorder among female police officers is noteworthy. Overall, the literature suggests that this finding requires further research, as the gender variable may change according to the type of incident experienced and its frequency [18]. In a study of prevalence among Dutch police officers, females were found to report more PTSD symptoms than males and, in association, they also reported having experienced life-threatening situations more frequently than their colleagues [43]. Similarly, in a cross-sectional survey of 359 American police officers, Hartley et al. [38] found that a higher frequency of traumatic events was associated with a higher prevalence of PTSD in females, while recent testimonies from victims of aggression were associated with a higher prevalence of PTSD in males. When comparing the incidence of traumatic events in this sample, females were found to report the experience of “having a family member and friend who had suffered violence” considerably more than males, (χ2 (df = 1, N = 3554) = 5.61; *p* < 0.05), indicating the presence of significant secondary exposure for this group. However, organizational factors such as interpersonal relationships in the work environment, harassment, and other impacting work-related critical incidents specially experienced by female officers in Rio de Janeiro should be better investigated in future research.

Additionally, in the military police of Rio de Janeiro, position in the hierarchy appears to be a predictor of the disorder, as soldiers, corporals, and also sub-lieutenants and sergeants are equally vulnerable to PTSD, unlike the officers in command positions. In fact, this conclusion is understandable in relation to soldiers and corporals since these are the military ranks most involved in the direct repression of crime, and, consequently, more exposed to critical incidents in their daily work. However, as far as sub-lieutenants and some sergeants, (ranks responsible for a unit’s supervision, training, and disciplinary control, also acting as a liaison between subordinates and upper management) are concerned, this finding may at first appear inconsistent, indicating that factors other than current exposure to critical incidents at work may be at stake for all these groups.

A possible explanation for this phenomenon may be related to the organizational factors to which these professionals, in subordinate duties, are subject. National studies carried out with police officers have attempted to identify the socio-occupational characteristics of the military police officers in Rio de Janeiro and have asserted how their working conditions directly impact their physical and mental health and contribute to chronic stress [44]. The unhealthy locations in which they perform their duties, the excessive workload and the constant lack of human resources are some of the factors that have contributed to the high level of mental suffering observed among military officers, especially among the lower military ranks. In addition, in military organizations, hierarchy is often highly valued and discipline is maintained through strict control and surveillance mechanisms. Such features can contribute to the distance among hierarchical cycles, and to the reduction of trust and mutual cooperation among professionals, which are fundamental factors in operations with a high potential for the occurrence of critical incidents [45]. Evidence in the literature suggests that the lack of social support and low levels of trust in interpersonal relationships observed in some police institutions are considered risk factors for mental suffering among these professionals [44,46].

Therefore, it is not an exaggeration to state that chronic work stress may contribute to greater susceptibility to PTSD, since the ability to cope with a critical incident may be reduced as a result of a pre-existing mental health condition, which exhausts and limits the professional’s individual resources to deal adequately with extreme situations.

It is also important to add that sub-lieutenants and sergeants joined the corporation as soldiers, and although many of them may now have administrative or supervisory responsibilities, they will have performed direct operational duties for many years, implying repeated re-exposure to threatening experiences, a factor considered as relevant as the traumatic magnitude of the event itself [1]. The accumulation of chronic stress caused by working conditions and exacerbated and repetitive exposure to violence may present an increased risk of PTSD for these police officers. However, given the improvement in the administrative management of the Military Police in recent years, further studies addressing the relationship between working conditions and chronic stress, and its effect on vulnerability to PTSD among military police officers are essential.

When observing the duties variable in this study, the police officers working in the administrative activity were found to be the most affected by the disorder. There may be two possible explanations for this finding. Firstly, one may assume that, although they perform internal duties, these police officers may have been exposed to traumatic experiences outside their working hours which may have led to the development of the disorder. A second way of understanding this phenomenon is to consider the institutional practice of transferring police officers who present some type of emotional instability from operational to administrative duties. This finding makes it possible to infer that, in many cases, the disorder may continue in the long term, even when the police officer is no longer a part of the operational scene. In fact, Kessler et al. [47] observed in The National Comorbidity Survey that in cases of PTSD lasting more than one year, remission would be unusual. Therefore, programs aimed at identifying these cases and referring them to specialized treatment should be reinforced.

In contrast, professionals with GAT/PATAMO duties, corresponding to tactical policing activities in urban areas, appear to be significantly less likely to suffer from PTSD. This finding contradicts the hypotheses advanced in this study that operational police officers, as they are more exposed to critical incidents in their daily work, would be at more risk to develop the disorder. This may indicate that, despite their greater exposure, these groups may have more protective factors, whether organizational (institutional social support, team cohesion, more training) or individual (resilience or hardiness, for example). Moreover, these police officers are more highly regarded professionals within the scope of the institution; however, the impact of this fact on the development of PTSD is yet to be evaluated. It is important to note that this finding is in line with the assumption that, in addition to exposure to critical events, organizational factors such as those described above, even in internal activities, may contribute to vulnerability to PTSD, and should be further investigated.

Finally, the work scale variable may contribute to PTSD. Although it was not possible to conclude which would be the best work scale to prevent full PTSD, the 24-h work shift schedule, with a 72 h rest period, showed a negative correlation with partial PTSD, suggesting that this variable needs to be deepened in order to clarify whether work shifts can, in fact, offer greater protection against the disorder.

Unlike some studies that have pointed to reduced professional experience increasing the risk of PTSD among young police officers [29,31], especially for those facing critical incidents for the first time, in the military police officers of Rio de Janeiro, age and tenure were not significant variables to predict the disorder. In this case, vulnerability extends to both younger and less experienced professionals and to older and more experienced officers, suggesting that the time period corresponding to experience does not appear to be an overriding protective factor in this case.

## 5. Conclusions

The results presented in this study clearly indicate that preventive actions aimed at police officers in operational policing activities should be reinforced and foster early interventions to prevent a worsening of the syndrome, considering the typical re-exposure experienced by lower ranks military personnel. It is already known that when PTSD symptoms are treated early, subsequent reactions to critical incidents are less extreme and less debilitating [48].

Moreover, as the results above indicate that not only critical exposure contributes to vulnerability to PTSD among Rio de Janeiro’s police officers, organizational practices and their role in predicting the disorder must be taken into account. It has been proven that certain organizational and managerial practices can mitigate the impact of on-duty trauma, through changes in culture, workload, institutional supervision style, and available social support [48]. Therefore, preventive actions should seek to mobilize various institutional entities in addition to health professionals. Such actions involve preventive forced leave after an incident, a rotation of services or areas of action, in addition to the adoption of more spaced out or staggered schedules for professionals involved in high-impact occurrences.

Furthermore, special attention should be paid to the police officers in the afore-mentioned risk range, that already suffer from PTSD, with the implementation of sound and more diversified mental health care measures. As described in the literature, the perception of available social support and satisfaction with the support received are protective factors against PTSD symptoms among police officers [49]. The identification of social support sources (institutional, family, health care, among others), acknowledged as reliable by professionals may be an important channel through which police officers who are resistant to traditional methods of care may be approached.

Future research is necessary to better explore the most harmful traumatic events to female police officers, as well as organizational factors that may contribute to their greater vulnerability. Additionally, research including work related variables, such as institutional support, management practices and team support, as well as individual factors (i.e., personality traits, resilience) that can deepen the analysis of PTSD risk among Rio de Janeiro military police officers are recommended.

Some of the limitations of this study need to be addressed. The gold standard for the diagnosis of PTSD is the semi-structured interview, which was not used in this research, and self-report measures are susceptible to some bias, such as underreporting, for example. There is a sampling disparity between males and females, commonly observed among military populations, which may reduce the possibility of adequately detecting the effect of this variable on PTSD in these groups. Furthermore, cumulative exposure and analysis of personal psychological variables were not addressed in this study. This is a cross-sectional study, and due to the limitations in establishing cause and effect analyses, longitudinal ones should also be carried out to better portray PTSD’s chronic trajectories in military police officers. Finally, given the specific features of this Military Police organization (structure and mission) and also the characteristics of the criminal dynamics of the state of Rio de Janeiro, the results of this study may not be generalizable to other contexts.

## Figures and Tables

**Table 1:** Relationships among post-traumatic stress symptoms status and socio-demographic and work-related variables.

Characteristics	No Post-traumatic Stress Disorder (PTSD)	Full PTSD	Group Comparison ^a^	Multivariate Analysis ^c^		
	(*n* = 2949)	(*n* = 605)	X^2^	Wald	B	OR (95% IC)
Gender ^b^			27.72 *	6.410 *		
Masculine	94.3%	88.4%			−0.485 *	0.616 (0.423–0.896)
Feminine ^d^	5.7%	11.6%				
Age ^c^			8.61 **	0.896		
[21–29 years old]	8.8%	7.5%			−0.220	0.802 (0.328–1.966)
[30–39 years old]	52.2%	47.7%			−0.126	0.882 (0.400–1.944)
[40–49 years old]	35.6%	41.9%			0.030	1.030 (0.504–2.106)
[50–58 years old] ^d^	3.3%	3.0%				
Education ^c^			22.60 **	2.793		
9th Degree	0.8%	1.2%			−0.280	0.756 (0.051–11.205)
12th Degree Uncompleted	0.4%	0.3%			−1.327	0.265 (0.010–6.923)
12th Degree Completed	64.0%	58.0%			−0.861	0.423 (0.035–5.165)
Bachelor degree Uncompleted	13.8%	19.8%			−0.518	0.596 (0.048–7.347)
Bachelor degree Completed	19.1%	19.5%			−0.797	0.451 (0.037–5.539)
Post-graduation	1.9%	0.8%			−1.448	0.235 (0.016–3.401)
Master degree ^d^	0.1%	0.3%				
Marital status ^c^			0.5.05	2.793		
Single	21.8%	21.6%			−0.436	0.647 (0.342–1.222)
Married	69.4%	67.2%			−0.457	0.633 (0.343–1.167)
Divorced	6.5%	7.5%			−0.284	0.753 (0.375–1.511)
Widower	0.3%	0.7%			−0.431	0.650 (0.143–2.948)
Other ^d^	2.0%	3.1%				
Tenure ^c^			15.06 **	7.647		
Less than 1 year	1.1%	0.5%			−0.089	0.914 (0.135–6.180)
Between 2 to 3 years	5.2%	3.7%			0.450	1.568 (0.339–7.244)
Between 4 to 5 years	17.4%	12.8%			0.085	1.089 (0.250–4.740)
Between 6 to 10 years	28.4%	28.9%			0.390	1.477 (0.346–6.304)
Between 11 to 19 years	33.6%	38.3%			0.665	1.945 (0.484–7.812)
Between 20 to 29 years	13.3%	15.1%			0.487	1.627 (0.420–6.308)
More than 30 years ^d^	1.0%	0.7%				
Position in the hierarchy ^c^			15.17 *	11.206 *		
Corporals and soldiers	54.5%	51.1%			1.707 *	5.515 (1.997–15.229)
Sergeants	36.5%	41.1%			1.359 *	3.893 (1.436–10.557)
Under-lieutenants	5.6%	6.9%			1.377 *	3.952 (1.382–11.299)
Officers ^d^	3.5%	1.0%				
Job function ^c^			123.71 *	18.281 *		
GAT/PATAMO	16.7%	8.4%			−0.507 *	0.602 (0.406–.892)
AI	2.4%	2.2%			−0.080	0.923 (0.471–1.807)
PO/APREV/RP	30.1%	19.1%			−0.352	0.703 (0.489–1.011)
ADM	13.8%	30.3%			0.417 *	1.518 (1.085–2.124)
Other	36.7%	39.7%				
Work schedule ^c^			111.46 *	25.015 *		
Workday from Monday to Friday	18.7%	35.3%			0.361	1.435 (0.396–5.193)
12 × 36	11.0%	11.2%			−0.049	0.952 (0.263–3.447)
12 × 24/12 × 48	29.9%	20.0%			−0.129	0.879 (0.242–3.194)
12/48	1.1%	2.4%			0.588	1.800 (0.419–7.728)
12/60	1.9%	2.6%			0.557	1.746 (0.424–7.193)
24/48	13.5%	15.9%			0.310	1.364 (0.381–4.875)
24/72	23.3%	11.9%			−0.510	0.601 (0.165–2.180)
48/96 ^d^	0.7%	0.7%				

Notes: ^a^ All *p*-values refer to the comparisons between “full” vs. “no PTSD” groups. ^b^ Chi-square test. ^c^ Wald, B (unstandardized regression coefficient), OR (odds ratio) and CI (confidence interval) refer to data from multivariate analysis through logistic regression. All predictor variables were transformed on dummy variables. ^d^ Comparative group. * *p* < 0.01; ** *p* < 0.05. GAT/PATAMO—Tactical Action Group—Special policing activities, usually carried out in high-risk locations and in critical situations, AI—Intelligence Agency—police intelligence duties, PO/APREV/RP—ordinary policing activity, ADM—secretariat, treasury, logistics, communication, and personnel control activities. To better understand the work schedules adopted, the first number must be considered the one corresponding to hours worked and the second to hours off. For instance, “12 × 36” corresponds to 12 h worked and 36 h off.

**Table 2:** Relationships among partial post-traumatic stress symptoms status and socio-demographic and work-related variables.

Characteristics	No PTSD	Partial PTSD	Group Comparison ^b^	Multivariate Analysis ^c^		
	(*n* = 2949)	(*n* = 952)	X^2^	Wald	B	OR (95% IC)
Gender ^b^			31.86 *	8.767 *		
Masculine	94.3%	89.4%			−0.516 *	0.597 (0.424–0.840)
Feminine	5.7%	10.6%				
Age ^c^			10.08 **	6.08		
[21–29 years old]	8.8%	9.0%			0.546	1.726 (0.790–3.774)
[30–39 years old]	52.2%	47.7%			0.197	1.217 (0.598–2.479)
[40–49 years old]	35.6%	40.6%			0.351	1.420 (0.742–2.717)
[50–58 years old]	3.3%	2.7%				
Education ^c^			18.75 **	7.983		
9th Degree	0.8%	0.8%			−0.523	0.593 (0.038–9.275)
12th Degree Uncompleted	0.4%	0.2%			−10.488	0.226 (0.008–6.125)
12th Degree Completed	64.0%	59.6%			−0.474	0.622 (0.048–8.050)
Bachelor degree Uncompleted	13.8%	18.5%			−0.168	0.845 (0.065–11.014)
Bachelor degree Completed	19.1%	19.6%			−0.366	0.694 (0.053–9.024)
Post-graduation	1.9%	1.2%			−0.669	0.512 (0.036–7.337)
Master degree	0.1%	0.2%				
Marital status ^c^			6.240	2.41		
Single	21.8%	21.2%			−0.215	0.806 (0.451–1.441)
Married	69.4%	68.0%			−0.174	0.841 (0.480–1.473)
Divorced	6.5%	7.4%			−0.019	0.981 (0.521–1.847)
Widower	0.3%	0.8%			0.431	1.538 (0.417–5.679)
Other	2.0%	2.6%				
Tenure ^c^			13.10 **	5.642		
Less than 1 year	1.1%	0.9%			0.212	1.236 (0.282–5.421)
Between 2 to 3 years	5.2%	3.5%			−0.367	0.693 (0.190–2.521)
Between 4 to 5 years	17.4%	14.6%			−0.177	0.838 (0.245–2.840)
Between 6 to 10 years	28.4%	28.9%			0.058	1.060 (0.314–3.580)
Between 11 to 19 years	33.6%	37.1%			0.145	1.156 (0.361–3.697)
Between 20 to 29 years	13.3%	14.3%			0.093	1.097 (0.353–3.413)
More than 30 years	1.0%	0.8%				
Position in the hierarchy ^c^			21.57 *	21.025 *		
Corporals and soldiers	54.5%	52.3%			10.999 *	7.380 (3.086–17.648)
Sergeants	36.5%	40.8%			1.820 *	6.173 (2.610–14.599)
Under-lieutenants	5.6%	5.9%			10.569 *	4.803 (1.930–11.951)
Officers	3.5%	1.0%				
Job function ^c^			142.02 *	24.959 *		
GAT/PATAMO	16.7%	10.2%			−0.345 *	.708 (0.521–0.963)
AI	2.4%	2.6%			0.262	1.299 (0.751–2.248)
PO/APREV/RP	30.1%	20.9%			−0.340 *	0.712 (0.527–0.962)
ADM	13.8%	27.8%			0.518 *	1.679 (1.245–2.265)
Other	36.7%	38.2%				
Work schedule ^c^			123.35 *	33.400 *		
Workday from Monday to Friday	18.7%	32.0%			−0.170	0.844 (0.316–2.255)
12 × 36	11.0%	11.4%			−0.554	0.575 (0.216–1.532)
12 × 24/12 × 48	29.9%	21.8%			−0.623	0.536 (0.201–1.430)
12/48	1.1%	2.2%			0.122	1.130 (0.351–3.632)
12/60	1.9%	2.0%			−0.197	0.821 (0.265–2.548)
24/48	13.5%	16.1%			−0.199	0.820 (0.311–2.162)
24/72	23.3%	13.6%			−0.995 *	0.370 (0.139–0.983)
48/96	0.7%	0.9%				

Notes: ^b^ Chi-square test. ^c^ Wald, B (unstandardized regression coefficient), OR (odds ratio) and CI (confidence interval) refer to data from multivariate analysis through logistic regression. All predictor variables were transformed on dummy variables. * *p* < 0.01; ** *p* < 0.005. GAT/PATAMO—Tactical Action Group—Special policing activities, usually carried out in high-risk locations and in critical situations, AI—Intelligence Agency—police intelligence duties, PO/APREV/RP—ordinary policing activity, ADM—secretariat, treasury, logistics, communication, and personnel control activities. To better understand the work schedules adopted, the first number must be considered the one corresponding to hours worked and the second to hours off. For instance, “12 × 36” corresponds to 12 h worked and 36 h off.

## Data Availability

The data presented in this study are available on request from the corresponding author.
